# De-normalizing sugar-sweetened beverage consumption: effects of tax measures on social norms and attitudes in the California Bay Area

**DOI:** 10.1186/s12889-024-20781-6

**Published:** 2024-11-25

**Authors:** Emily Altman, Dean Schillinger, Sofia Villas-Boas, Laura Schmidt, Jennifer Falbe, Kristine A. Madsen

**Affiliations:** 1 School of Public Health, University of California, 2121 Berkeley Way, Berkeley, CA 94720 USA; 2 School of Medicine, University of California, San Francisco, CA USA; 3 Department of Agricultural and Resource Economics, University of California, Berkeley, CA USA; 4 Philip R Lee Institute for Health Policy Studies, University of California, San Francisco, CA USA; 5 Department of Humanities and Social Sciences, University of California, San Francisco, CA USA; 6 Human Development and Family Studies Program, Department of Human Ecology, University of California, Davis, CA USA

**Keywords:** Sugar-sweetened beverages, Public health policy, Social norms, Health attitudes

## Abstract

**Background:**

Social norms can influence individual health behaviors. Shifts in social norms for smoking were critical for the effectiveness of tobacco control efforts such as excise taxes. Sugar-sweetened beverage (SSB) excise taxes have been implemented in municipalities across the United States to reduce SSB intake and improve health. We sought to identify trends in social norms and attitudes about healthfulness of sugar-sweetened beverage (SSB) consumption in the California Bay Area and examine whether social norms and attitudes changed following SSB taxes.

**Methods:**

Data came from annual (2016–2019, 2021) cross-sectional surveys (*n* = 9128) in lower-income neighborhoods in Oakland, San Francisco, Berkeley, and Richmond. We assessed overall trends and compared pre-post tax changes in Oakland and San Francisco with comparison cities.

**Results:**

We observed a 28% reduction in social norms for SSB consumption (people’s perceptions of peers’ consumption) and variable reductions in attitudes about the healthfulness of SSBs. Relative to comparison cities, post-tax, perceptions of peers’ consumption of sports drinks declined in Oakland; attitudes about the healthfulness of sugar-sweetened fruit drinks declined in San Francisco.

**Conclusions:**

Among lower-income populations, social norms and attitudes towards the healthfulness of SSBs meaningfully declined over time, with smaller tax-related effects. SSB taxes as well as the local media attention they generate appear to affect people’s perceptions of SSBs. Pairing SSB taxes with messaging campaigns may be more effective in de-normalizing SSB consumption.

**Supplementary Information:**

The online version contains supplementary material available at 10.1186/s12889-024-20781-6.

## Background

Social norms, or people’s perceptions of what is socially acceptable, are important as they influence people’s behavior via informal rules for how people should behave—independent of taxes and other financial disincentives (i.e., price changes) [1, 2]. Both social norms and attitudes are known to impact dietary behaviors; social norms (individuals’ perceptions of other people’s dietary and SSB intake) have been shown to be related to people’s own dietary consumption [3–7], and shifts in people’s attitudes about the healthfulness of SSBs also predict shifts in their consumption [8, 9]. Within the tobacco control movement, the concept of ‘de-normalizing’ tobacco use has been fundamental to the movement’s success. In fact, implementing tobacco taxes alongside messaging campaigns, such as social marketing, has shifted social norms over multiple decades [10–12].

In recent years, SSB excise taxes have been implemented in municipalities across the United States as a tool to reduce SSB intake and improve health [13, 14]. Multiple studies analyzing SSB sales data have reported declines in sales following the implementation of SSB taxes [15]. In terms of social norms and attitudes around SSBs, while public health advocates may hope to see changes in social norms, de-normalizing SSB consumption has not been emphasized consistently in cities with these taxes. Shifting norms and attitudes may be an opportunity to further improve health via SSB taxes.

Because taxes proposed via ballot measure are accompanied by significant advertising and media, social norms and attitudes could shift in response to tax campaigns. A study conducted using sales data from the University of California, Berkeley found that SSB sales declined on the college campus after the city’s SSB tax was passed via ballot measure but before SSB prices increased [16]. Another study conducted following Seattle’s SSB tax found that perceptions of the health risks of consuming SSBs increased post-tax for lower-income respondents [17]. These findings may indicate that tax campaign messaging and the taxes themselves can influence short-term behavior by changing social norms and attitudes, independent of price changes, though further research is needed to better understand the relationship between SSB taxes and shifts in social norms and attitudes.

Understanding whether SSB-related social norms and attitudes have shifted over time, as well as in the short-term following SSB taxes, could help policymakers and public health advocates identify opportunities to de-normalize SSB consumption and potentially strengthen the public health impacts of SSB taxes. The primary aim of the current study was to assess, from 2016–2021 whether social norms and attitudes related to SSBs have shifted over time across the California Bay Area. In an exploratory analysis, we also aimed to assess whether social norms and attitudes changed following election-related media campaigns and implementation of SSB taxes in Oakland and San Francisco, CA relative to nearby comparison cities (Berkeley, which had a pre-existing SSB tax, and Richmond, which had no SSB tax). Considering that lower-income populations experience frequently targeted marketing of SSBs and have a greater burden of diet-related disease, the current study focuses on populations living in lower-income neighborhoods [18–20].

## Methods

### California bay area SSB taxes

**Fig. 1:**
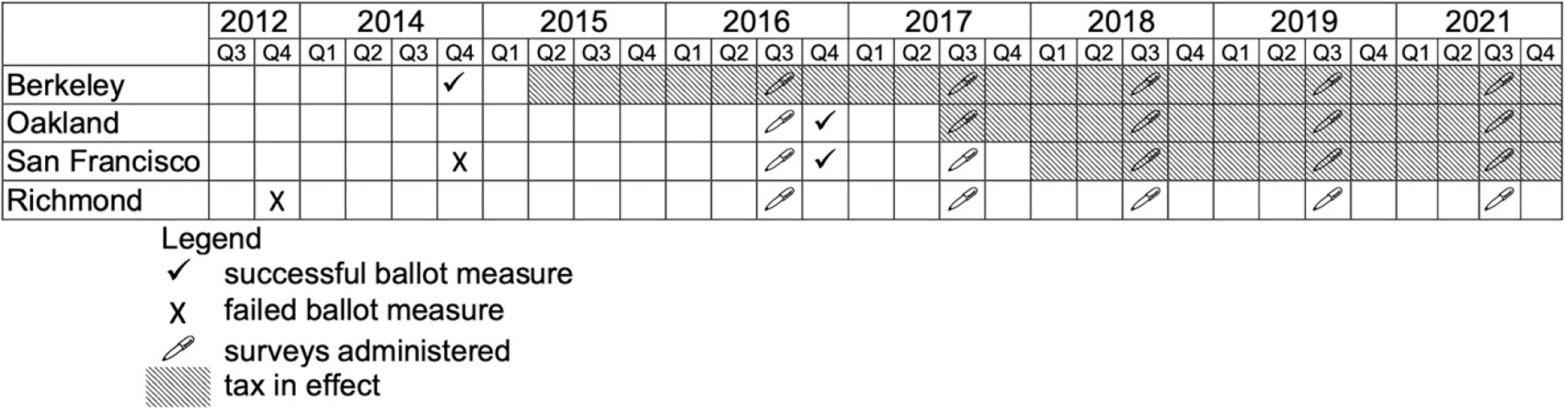
Timeline of SSB ballot measures, tax implementation, and study data collection, 2012–2021

Between 2014–2016, multiple cities in the California Bay Area voted on and passed SSB taxes. Berkeley voted on and passed an SSB tax in November 2014 (implemented in March 2015). In November 2016, Oakland voted on and passed an SSB tax (implemented in July 2017). San Francisco also passed an SSB tax in November 2016 (implemented in January 2018). Ballot measures were previously held but proved to be unsuccessful in Richmond (2012) and San Francisco (2014) (Fig. 1). Over this time period, the Bay Area was exposed to a significant and unprecedented amount of SSB-related media coverage, including messages related to the hazards of SSB consumption [21]. The present study was conducted against the backdrop of this changing media and policy landscape.

### Study design & participants

Using a serial cross-sectional study design, we conducted annual street intercept surveys with Berkeley, Oakland, San Francisco, and Richmond, CA residents between 2016–2019 and 2021. Street intercept surveys were not conducted in 2020 due to the COVID-19 pandemic. We sampled participants from demographically diverse neighborhoods in each city. Neighborhood selection and details about the survey administration have been presented previously [22]: using 2010 census data, we identified two neighborhoods in each city with a high proportion of Hispanic/Latinx, Black, and low-income residents. Trained research assistants conducted intercept surveys with participants on busy street corners in those neighborhoods. This study was considered exempt by the Committee for Human Subjects and verbal consent was obtained from all survey respondents.

### Measures

We assessed social norms around SSB consumption using a modified beverage frequency questionnaire (BFQ) [23], asking participants, “How often do you think other people your age in [your city] drink…” separately for soda, sports drinks, and fruit drinks. To assess attitudes about the healthfulness of SSBs, we used the following question separately for soda, sports drinks, and fruit drinks: “How healthy do you think the following drinks are, on a scale of 1 (extremely unhealthy) to 7 (extremely healthy)?” Both the social norms (perceived consumption) and attitudes items were based on literature exploring SSBs and the Theory of Planned Behavior [8].

Participants were also asked about their race/ethnicity (Asian, Black, Hispanic/Latinx, White, Other), education (less than high school, high school/GED, some college, college graduate or higher), gender, age, and their own SSB consumption.

### Statistical analyses

To examine trends in social norms, or perceived SSB consumption, we calculated the adjusted marginal predicted frequency of other people’s soda, sports drink, and fruit drink consumption (i.e., respondents’ perception of what their peers consume) using a Tobit model to censor outcomes at 0 and to account for the high proportion of expectations of no consumption and robust standard errors. We adjusted for respondent gender, age, race/ethnicity, education, SSB consumption, and neighborhood. Although we might expect that respondents’ SSB consumption may be linked with the outcomes assessed, controlling for SSB consumption removes the potential confounding effect of personal consumption habits on perceived social norms or attitudes. To assess trends over time across all cities, as well as individually by city, we used year (continuous) as the primary predictor and use Stata’s lincom command to estimate 5-year declines from 2016 to 2021. The value for 2021 was treated as two years after 2019 (i.e., t + 2 relative to 2019). In the exploratory analysis assessing the additional association between SSB taxes and norms, we used a comparison-of-differences analysis that provides the adjusted difference in change in pre- versus post-tax periods in newly taxed cities (Oakland and San Francisco) versus comparison cities (Richmond and Berkeley), using an interaction term between newly taxed city and a binary indicator for tax implementation. In San Francisco, we also had the opportunity to assess changes following the ballot measure passage, prior to tax implementation, because we collected data in fall 2017, after the 2016 ballot measure but prior to 2018 tax implementation. As such, we also assessed the association with the ballot measure in San Francisco by adding an interaction term between city and a binary indicator for the year the ballot measure passed.

To examine attitudes about the healthfulness of SSBs, we used the same techniques as for social norms but calculated adjusted predicted attitudes using linear models.

Comparison cities included Berkeley (whose tax was implemented in 2015, before the start of this analysis) and Richmond (not taxed but exposed to media coverage). Because Berkeley’s tax status was consistent over the study period, we assumed that social norms would not be affected by new taxes in Oakland and San Francisco but might be affected by associated media coverage. Because Richmond is in the same media market as Oakland and San Francisco, it is possible that residents of Richmond were newly exposed to messaging from SSB tax ballot measures in Oakland and San Francisco as their ballot measures were being voted on or implemented. To explore this potential contamination, we carried out a sensitivity analysis in which we dropped Richmond as a comparison and compared changes in Oakland and San Francisco with changes in just Berkeley.

In the additional files, we include a table comparing city-wide and study population demographic characteristics, as well as graphs showing adjusted estimates of social norms year by year, by city. Per the literature, this analysis did not adjust for multiple comparisons [24]. All analyses were conducted in Stata/SE 16.1 (StataCorp LLC, College Station, TX, USA).

## Results

The sample included 9,128 respondents across the 2016–2019 and 2021 data collection periods. Most respondents identified as Black (32%) or Hispanic/Latinx (33%) and 60% had at least some college education or more. Berkeley’s sample included more respondents who identified as White and had a college education compared to Oakland, San Francisco, and Richmond (see Table 1).

**Table 1:** Respondent characteristics by city, 2016–2019 and 2021

*n* (%)	Total	Berkeley	Oakland	San Francisco	Richmond
	***N* = 9,128**	***N* = 1,877**	***N* = 2,568**	***N* = 2,377**	***N* = 2,306**
Years of interview					
2016	1,689 (19%)	333 (18%)	525 (20%)	442 (19%)	389 (17%)
2017	2,144 (23%)	360 (19%)	616 (24%)	594 (25%)	574 (25%)
2018	2,489 (27%)	471 (25%)	724 (28%)	653 (27%)	641 (28%)
2019	1,626 (18%)	392 (21%)	434 (17%)	392 (16%)	408 (18%)
2021	1,180 (13%)	321 (17%)	269 (10%)	296 (12%)	294 (13%)
Race/ethnicity					
Black	2,950 (32%)	416 (22%)	1,081 (42%)	591 (25%)	862 (37%)
Hispanic/Latinx	3,007 (33%)	270 (14%)	1,018 (40%)	976 (41%)	743 (32%)
White	1,835 (20%)	810 (43%)	189 (7%)	449 (19%)	387 (17%)
Asian	651 (7%)	208 (11%)	99 (4%)	187 (8%)	157 (7%)
Other	685 (8%)	173 (9%)	181 (7%)	174 (7%)	157 (7%)
Education					
< High school	1,380 (15%)	65 (3%)	626 (24%)	448 (19%)	241 (10%)
High school	2,279 (25%)	304 (16%)	760 (30%)	575 (24%)	640 (28%)
Some college	2,334 (26%)	413 (22%)	680 (26%)	535 (23%)	706 (31%)
College grad +	3,135 (34%)	1,095 (58%)	502 (20%)	819 (34%)	719 (31%)
Gender					
Male	4,082 (45%)	916 (49%)	1,032 (40%)	1,098 (46%)	1,036 (45%)
Female	4,988 (55%)	949 (51%)	1,520 (59%)	1,255 (53%)	1,264 (55%)
Other	58 (1%)	12 (1%)	16 (1%)	24 (1%)	6 (0%)
Age					
18–29	2,275 (25%)	507 (27%)	658 (26%)	479 (20%)	631 (27%)
30–39	1,625 (18%)	307 (16%)	430 (17%)	468 (20%)	420 (18%)
40–49	1,407 (15%)	247 (13%)	454 (18%)	409 (17%)	297 (13%)
50–59	1,654 (18%)	276 (15%)	477 (19%)	513 (22%)	388 (17%)
≥ 60	2,167 (24%)	540 (29%)	549 (21%)	508 (21%)	570 (25%)
SSB consumption times/day (mean, SD)	1.11 (1.76)	0.62 (1.30)	1.44 (1.90)	1.00 (1.59)	1.27 (1.97)

### Social norms

Baseline values for social norms and attitudes about the healthfulness of SSBs by city are detailed in Table 2. Across all cities combined, respondent perceptions of their peers’ consumption of SSBs declined significantly over the period under study, with an average annual decline of −0.11 times/day (95% CI: −0.13, −0.08) for soda (representing a 28% decline over the 5 years from 2016 to 2021), −0.05 times/day (95% CI: −0.08, −0.03) for sports drinks (a 26% decline), and −0.07 times/day (95% CI: −0.09, −0.05) for fruit drinks (a 28% decline). Models at the city level also demonstrated significant annual declines in each of the four cities in perceived consumption of soda (5-year declines ranged from 24 to 32%), sports drinks (5-year declines from 22 to 30%), and fruit drinks (5-year declines from 17 to 40%) across the data collection period (Table 3). The largest declines in perceived consumption of SSBs were observed for those cities that had the greatest perceived consumption of SSBs at baseline (Table 2).

**Table 2:** Adjusted^a^ marginal baseline (2016) values for social norms and attitudes about the healthfulness of SSBs by city

	**Perceived consumption**^**b**^** (mean, SE)**		
	**Soda**	**Sports drinks**	**Fruit drinks**
Overall	1.85 (0.04)	1.06 (0.04)	1.23 (0.04)
By city			
Berkeley	1.53 (0.09)	1.00 (0.08)	1.09 (0.08)
Oakland	2.12 (0.09)	1.47 (0.09)	1.43 (0.10)
San Francisco	1.69 (0.09)	0.92 (0.08)	1.03 (0.07)
Richmond	2.04 (0.09)	0.90 (0.08)	1.33 (0.09)
	**Attitudes about healthfulness**^**c**^** (mean, SE)**		
	**Soda**	**Sports drinks**	**Fruit drinks**
Overall	1.75 (0.03)	3.17 (0.04)	3.15 (0.04)
By city			
Berkeley	1.64 (0.07)	2.85 (0.09)	2.86 (0.09)
Oakland	1.86 (0.07)	3.24 (0.08)	3.30 (0.08)
San Francisco	1.71 (0.06)	3.26 (0.08)	3.28 (0.08)
Richmond	1.77 (0.07)	3.26 (0.09)	3.08 (0.09)

^a^Models adjust for gender, age, race/ethnicity, education, and SSB consumption

^b^Reflects how often respondents think others consume said beverages, times/day

^c^Reflects respondents’ attitudes about the healthfulness of said beverages on a scale from 1 (extremely unhealthy) to 7 (extremely healthy)

**Table 3:** Adjusted annual trends in perceived consumption and attitudes about the healthfulness of SSBs

City	Adjusted annual trends in perceived consumption,^a^ times per day (95% CI)		
	**Soda**	**Sports drinks**	**Fruit drinks**
Overall	**−0.11 (−0.13, −0.08)**^**c**^	**−0.05 (−0.08, −0.03)**^**c**^	**−0.07 (−0.09, −0.05)**^**c**^
By city			
Berkeley	**−0.09 (−0.12, −0.05)**^**c**^	**−0.05 (−0.08, −0.01)**^**c**^	**−0.04 (−0.07, −0.00)**^**c**^
Oakland	**−0.12 (−0.17, −0.08)**^**c**^	**−0.09 (−0.13, −0.04)**^**c**^	**−0.09 (−0.14, −0.05)**^**c**^
San Francisco	**−0.08 (−0.12, −0.04)**^**c**^	**−0.04 (−0.08, −0.00)**^**c**^	**−0.04 (−0.08, −0.00)**^**c**^
Richmond	**−0.13 (−0.17, −0.09)**^**c**^	**−0.04 (−0.08, −0.00)**^**c**^	**−0.11 (−0.15, −0.06)**^**c**^
**City**	**Adjusted annual trends in attitudes about the healthfulness of SSBs,**^**b**^** (95% CI)**		
	**Soda**	**Sports drinks**	**Fruit drinks**
Overall	0.00 (−0.02, 0.02)	−0.01 (−0.04, 0.01)	−0.01 (−0.03, 0.02)
By city			
Berkeley	0.02 (−0.02, 0.05)	0.03 (−0.01, 0.07)	0.03 (−0.01, 0.07)
Oakland	0.01 (−0.04, 0.05)	−0.01 (−0.06, 0.04)	−0.02 (−0.07, 0.03)
San Francisco	−0.01 (−0.05, 0.02)	**−0.06 (−0.11, −0.01)**^**c**^	**−0.08 (−0.13, −0.04)**^**c**^
Richmond	−0.00 (−0.04, 0.04)	−0.01 (−0.06, 0.04)	**0.05 (0.00, 0.10)**^**c**^

^a^Models adjust for gender, age, race/ethnicity, education, and SSB consumption, and neighborhood. Estimates reflect annual trends in perceived consumption (how often respondents think others consume said beverages, times/day)

^b^Models adjust for gender, age, race/ethnicity, education, and SSB consumption, and neighborhood. Estimates reflect annual trends in attitudes about the healthfulness of soda, sports drinks, and fruit drinks on a scale from 1 (not at all healthy) to 7 (very healthy)

^c^Results are significant; a 95% confidence interval does not include the null

Following tax implementation in Oakland and San Francisco and post-ballot in San Francisco, there were no additional significant changes in perceived consumption of soda relative to the other two comparison cities. In Oakland, respondents reported lower perceived sports drink consumption post-tax by an additional 0.46 times/day (95% CI: −0.69, −0.22; a 22% decline from baseline), compared with changes in Berkeley and Richmond. There were no significant changes for fruit drinks in Oakland or sports or fruit drinks in San Francisco due to the ballot passing or tax implementation relative to the comparison cities (Table 4).

**Table 4:** Adjusted annual change in perceived consumption and attitudes due to SSB tax, relative to comparison

City	Time period	Adjusted annual change in perceived consumption,^a^ times per day (95% CI)		
		**Soda**	**Sports drinks**	**Fruit drinks**
Oakland^b^	Tax implementation	−0.03 (−0.26, 0.19)	**−0.46 (−0.69, −0.22)**^**e**^	−0.12 (−0.35, 0.12)
San Francisco^c^	Ballot passing	0.18 (−0.07, 0.44)	0.05 (−0.20, 0.29)	0.20 (−0.04, 0.43)
	Tax implementation	0.17 (−0.05, 0.40)	−0.01 (−0.22, 0.21)	0.18 (−0.02, 0.38)
**City**	**Time period**	**Adjusted annual change in attitudes about healthfulness of SSBs**^**d**^** (95% CI)**		
		**Soda**	**Sports drinks**	**Fruit drinks**
Oakland^b^	Tax implementation	−0.10 (−0.27, 0.08)	−0.10 (−0.31, 0.12)	−0.05 (−0.27, 0.17)
San Francisco^c^	Ballot passing	−0.06 (−0.26, 0.14)	−0.09 (−0.35, 0.18)	−0.00 (−0.26, 0.27)
	Tax implementation	−0.10 (−0.27, 0.06)	−0.22 (−0.45, 0.01)	**−0.35 (−0.58, −0.12)**^**e**^

^a^Models adjust for gender, age, race/ethnicity, education, SSB consumption, and neighborhood. Estimates compare differences between newly taxed and comparison cities in perceived consumption (how often respondents think others consume said beverages, times/day)

^b^Oakland’s 2017–2021 post-tax implementation estimates are relative to 2016. Comparison cities include Berkeley and Richmond

^c^San Francisco’s 2017 post-ballot measure/pre-tax implementation estimates are relative to 2016, and the 2018–2021 estimates are the additional effect following the implementation of the tax. Comparison cities include Berkeley and Richmond

^d^Models adjust for gender, age, race/ethnicity, education, and SSB consumption, and neighborhood. Estimates compare differences between newly taxed and comparison cities in attitudes about the healthfulness of soda, sports drinks, and fruit drinks on a scale from 1 (extremely unhealthy) to 7 (extremely healthy)

^e^Results are significant; a 95% confidence interval does not include the null

Results from the sensitivity analysis that dropped Richmond as a comparison city yielded similar effect sizes, although the effect on fruit drinks post-ballot in San Francisco compared to just Berkeley (0.26, 95% CI: 0.01, 0.52) became statistically significant (see Additional File 1, Supplemental Table 1).

### Attitudes about health

Across all cities combined, there were no significant yearly trends in attitudes about the healthfulness of soda (0.00, 95% CI: −0.02, 0.02), sports drinks (−0.01, 95% CI: −0.04, 0.01), or fruit drinks (−0.01, 95% CI: −0.03, 0.02). There were some differences by city, with respondents in San Francisco reporting less favorable attitudes about the healthfulness of sports drinks by 0.06 units (95% CI: −0.11, −0.01; representing a 5-year decline of 10%) and fruit drinks by 0.08 units (95% CI: −0.13, −0.04; a 5-year decline of 13%) yearly over time. Respondents in Richmond reported an increase in attitudes about the healthfulness of fruit drinks across the study period by 0.04 units (95% CI: 0.00, 0.10; 5-year decline of 3%) (Table 3).

Following the implementation of its tax in 2018, respondents in San Francisco reported less favorable attitudes about the healthfulness of fruit drinks by 0.35 units (95% CI: −0.58, −0.12; 11% decline from baseline) compared with changes in Berkeley and Richmond. In San Francisco and Oakland, there were no other changes in attitudes about the healthfulness of any beverage post-tax implementation, or after ballot passage (Table 4).

Results from the sensitivity analysis dropping Richmond as a comparison city yielded similar results, although the change in attitudes about the healthfulness of soda in San Francisco following tax implementation (−0.34, 95% CI: –0.60, –0.08) became statistically significant (see Additional File 1, Supplemental Table 2).

## Discussion

In this study of changes in social norms and attitudes about the healthfulness of SSBs, we found that among lower-income communities, there were gradual and robust reductions in SSB-related social norms, with the greatest effects observed for those cities that had the least healthy social norms at baseline. We observed a decline in social norms (i.e., how frequently respondents believed their peers consumed SSBs) for soda, sports drinks, and fruit drinks across all cities. We also found variable shifts in attitudes about the healthfulness of SSBs: over time, respondents in San Francisco reported less favorable attitudes about the healthfulness of sports drinks and fruit drinks. In our exploratory analysis looking at how social norms and attitudes about the healthfulness of SSBs changed following SSB taxes, we found that following tax implementation in Oakland, respondents reported lower perceived consumption of sports drinks by their peers relative to changes in comparison cities. In San Francisco, respondents reported less favorable attitudes about the healthfulness of fruit drinks post-tax relative to changes in comparison cities.

Given the established influence of social norms/expectations [3–7] and health attitudes [8] on SSB intake, the shifting trends in social norms and attitudes we document across the California Bay Area over time are promising and align with the multiple studies that found declines in SSB sales following implementation of SSB taxes [15]. Our findings suggest that SSB consumption is becoming de-normalized, in cities with and without SSB taxes. Between 2014–2018, at a time when three of the four cities in our sample were exposed to SSB taxes, there were over 700 news stories related to SSB taxes across California [21]. This implies that our California Bay Area sample – including cities with and without SSB taxes – have been exposed to significant media coverage. Thus, the significant trends in social norms we observed across our sample likely represent a response to strong media coverage around SSB taxes and the health harms of SSB intake, combined with the direct effects of the taxes.

Over many years, the beverage industry has pushed a strong narrative that SSBs are normative components of daily life. Companies such as Coca-Cola and PepsiCo use a variety of methods to make their products ubiquitous, such as creating and selling memorabilia, selling personalized cans, and contracting with celebrities to advertise their products [25]. Our findings that some SSB-related social norms and attitudes have shifted over time in low-income neighborhoods in the California Bay Area suggests that SSB taxes and the attendant media may play important roles in countering beverage industry messaging.

Compared to social norms, we found more variable shifts in respondents’ attitudes about the healthfulness of SSBs over time across cities and moderate shifts as a result of the tax. It is likely that the limited change we observed in respondents’ attitudes about the healthfulness of soda was due to a floor effect; baseline perceptions of the unhealthfulness of soda may have left little room for further declines (see Table 2). The minimal shifts in attitudes about the healthfulness of sports and fruit drinks may reflect a lack of knowledge that these products contain added sugars, or the fact that people often perceive sports and fruit drinks to be the healthiest sugary drink options [26]. This suggests there may still be an opportunity to educate the public about the high sugar content in all SSBs and highlights the importance of de-normalizing consumption of sports and fruit drinks in particular.

When interpreting the association we documented in our exploratory analysis between SSB taxes and social norms, it is important to note that the comparison cities used in the present study, Berkeley and Richmond, are in the same media market as the taxed cities and were subject to their own SSB-tax measures previously (Richmond 2012 [failed]; Berkeley 2014 [passed]). Contamination from media in Richmond and from the ongoing tax in Berkeley would bias our results towards the null. Despite this bias, we did find declines in perceived consumption of sports drinks that appeared to be associated with the tax.

The relatively small associations we found between the tax and social norms and attitudes about healthfulness of SSBs may reflect the limitations of our comparison cities, but may also represent a missed public health opportunity to heighten awareness among consumers. Our prior study documented that in the year following tax implementation, fewer than 40% of a sample of lower-income residents in Oakland and San Francisco reported being aware that an SSB tax had been passed, while in Richmond, 14% of respondents incorrectly reported that a tax had been passed in their city [27]. These findings provide further evidence that social messaging is a critical adjunct to tax campaigns and should perhaps be one way that SSB tax revenue should be spent to maximize its benefit. In fact, tax revenues in Berkeley were used to fund media campaigns about healthy beverages [28]; this may serve as an example of a best practice for use of tax revenues in other cities with SSB taxes. Additionally, SSB taxes, which currently cover small geographies and represent relatively low effective tax rates (1 cent-per-ounce in the Bay Area), would likely lead to larger shifts in awareness and social norms if they were larger and covered larger geographies.

We believe it is useful to examine changes in SSB intake through the lens of tobacco control. While no single policy change has been causally attributed to a reduction in smoking prevalence, the combined effects over many decades of taxation and other policy interventions, along with ongoing public awareness and education campaigns of the health consequences of smoking, has contributed to a shift in social norms about the acceptability of smoking. Over a long period of time, this has led to a significant decline in smoking prevalence and its sequelae [11, 29–31]. Thus, we would expect that even the modest shifts in social norms and attitudes observed in our study can contribute to cumulative reductions in SSB intake at the population level, potentially leading to meaningful health benefits over time. Given the ubiquity of SSB advertising [25], employing multiple interventions at multiple levels (from individual to policy) across numerous years will undoubtedly be necessary to de-normalize SSB and added sugar consumption [32, 33]. Among the many lessons learned from the tobacco control movement, large changes in social norms are possible but require multi-faceted strategies to produce large, measurable impacts [11].

One concern about SSB taxes is the potential for smaller effects among lower-income populations [34, 35]. Our study, conducted in lower-income neighborhoods in the California Bay Area, revealed robust shifts in perceived consumption over time and more modest shifts specifically associated with SSB tax implementation. However, ours was only an exploratory analysis, and there is limited other research examining how social norms and attitudes have changed in the broader population following SSB taxes. Further research is warranted to better understand the impacts of SSB taxes on social norms among the general population and to identify additional interventions that might further shift social norms and attitudes.

This study has several limitations. Surveys relied on a convenience sample in four cities in the California Bay Area, potentially limiting the generalizability of our findings to other populations and cities. As noted, in analyses looking at the association between SSB taxes and social norms, the comparison cities were not ideal: there is potential for contamination because all cities in the study were exposed to media messaging and people may live in one city but work in another, both of which could dilute the observable differences attributable to SSB taxes, biasing the results towards the null. Also, while a traditional difference-in-differences analysis would be useful in this context, we may not have satisfied the parallel trends assumption in our analysis looking at the association between social norms and attitudes with SSB taxes, as pre-tax trends looked to be different across taxed versus comparison cities. Additionally, 2016 surveys were conducted before ballot measures were voted on, but media attention on SSB tax ballot measures was already present. Thus, respondents in the pre-tax comparison year may have been exposed to SSB tax messaging, which would also bias toward the null. Although we adjust for time and other potential confounding variables, we were not powered to assess for interaction. As in many observational studies, unmeasured confounding may affect our estimates.

## Conclusions

Our study documented promising shifts in SSB-related social norms across the California Bay Area over time, as well as a modest additional association between SSB taxes and social norms and attitudes in Oakland and San Francisco. Our findings suggest there is opportunity for multi-faceted strategies and additional interventions to shift social norms and attitudes further and de-normalize regular consumption of SSBs. In the future, public health messaging and campaigns should consider social norms as a powerful mechanism by which SSB taxes and other SSB control efforts impact behavior. Consideration should be given to harnessing SSB tax revenue to support public heath campaigns to further de-normalize SSB consumption. More research over longer periods of time is needed to understand trends in social norms over time, as well as the impact of SSB taxes on social norms and attitudes.

## Supplementary Information

Additional file 1: Supplemental Table 1. City-wide and study characteristics by city and year, 2016–2019 and 2021. Supplemental Table 2. Sensitivity analysis for adjusted change in perceived consumption of soda, sports drink, and fruit drink consumption (times per day), with Berkeley as a comparison city. Supplemental Table 3. Sensitivity analysis for adjusted change in attitudes about the healthfulness of soda, sports drinks, and fruit drinks, with Berkeley as a comparison city. Supplemental Fig. 1. Adjusted marginal social norms (perceived consumption) of SSBs (soda, sports drinks, and fruit drinks) by city and year (times/day). Supplemental Fig. 2. Adjusted marginal attitudes about healthfulness of soda, sports drinks, and fruit drinks, by city and year.

## Data Availability

The datasets generated and analyzed during the current study are available from the corresponding author by request. Data will be publicly available soon.

## Abbreviation

SSB: Sugar-sweetened beverage
