# Supplementary material for: De-normalizing sugar-sweetened beverage consumption: effects of tax measures on social norms and attitudes in the California Bay Area
Source: BMC Public Health. 2024 Nov 25;24:3263. doi: 10.1186/s12889-024-20781-6 (PMC11587705; doi:10.1186/s12889-024-20781-6)
Supplement: Supplementary file 1 — Additional file 1: Supplemental Table 1. City-wide and study characteristics by city and year, 2016–2019 and 2021. Supplemental Table 2. Sensitivity analysis for adjusted change in perceived consumption of soda, sports drink, and fruit drink consumption (times per day), with Berkeley as a comparison city. Supplemental Table 3. Sensitivity analysis for adjusted change in attitudes about the healthfulness of soda, sports drinks, and fruit drinks, with Berkeley as a comparison city. Supplemental Fig. 1. Adjusted marginal social norms (perceived consumption) of SSBs (soda, sports drinks, and fruit drinks) by city and year (times/day). Supplemental Fig. 2. Adjusted marginal attitudes about healthfulness of soda, sports drinks, and fruit drinks, by city and year. [file 12889_2024_20781_MOESM1_ESM.docx]

De-normalizing Sugar-Sweetened Beverage Consumption: Effects of Tax Measures on Social Norms and Attitudes in the California Bay Area

Supplemental Material

Supplemental Table 1. City-wide and study characteristics by city and year, 2016-2019 and 2021

| **2016** | | | **Berkeley** | | | | | | **Oakland** | | | | **San Francisco** | | | | **Richmond** | | |  |
| --- | --- | --- | --- | --- | --- | --- | --- | --- | --- | --- | --- | --- | --- | --- | --- | --- | --- | --- | --- | --- |
|  | | | City | | | Study | | | City | | Study | | City | | Study | | City | | Study |  |
| Race/ethnicity | | |  | | |  | | |  | |  | |  | |  | |  | |  |  |
| Black | | | 8% | | | 24% | | | 23% | | 47% | | 5% | | 26% | | 17% | | 42% |  |
| Hispanic/Latinx | | | 11% | | | 11% | | | 26% | | 36% | | 15% | | 43% | | 45% | | 31% |  |
| White | | | 55% | | | 49% | | | 29% | | 6% | | 41% | | 17% | | 18% | | 14% |  |
| Asian | | | 20% | | | 8% | | | 16% | | 5% | | 34% | | 6% | | 16% | | 5% |  |
| Other | | | 6% | | | 9% | | | 6% | | 6% | | 5% | | 8% | | 5% | | 7% |  |
| Education | | |  | | |  | | |  | |  | |  | |  | |  | |  |  |
| <High school | | | 4% | | | 4% | | | 18% | | 23% | | 12% | | 18% | | 22% | | 11% |  |
| High school | | | 10% | | | 16% | | | 16% | | 31% | | 13% | | 26% | | 23% | | 28% |  |
| Some college | | | 28% | | | 22% | | | 26% | | 27% | | 20% | | 24% | | 31% | | 33% |  |
| College grad + | | | 58% | | | 58% | | | 39% | | 19% | | 56% | | 32% | | 23% | | 29% |  |
| **2017** | | | **Berkeley** | | | | | | **Oakland** | | | | **San Francisco** | | | | **Richmond** | | |  |
|  | | | City | | | Study | | | City | | Study | | City | | Study | | City | | Study |  |
| Race/ethnicity | | |  | | |  | | |  | |  | |  | |  | |  | |  |  |
| Black | | | 8% | | | 25% | | | 23% | | 42% | | 5% | | 22% | | 18% | | 34% |  |
| Hispanic/Latinx | | | 11% | | | 13% | | | 26% | | 38% | | 15% | | 43% | | 48% | | 35% |  |
| White | | | 54% | | | 41% | | | 29% | | 10% | | 40% | | 20% | | 17% | | 17% |  |
| Asian | | | 21% | | | 11% | | | 16% | | 4% | | 34% | | 7% | | 13% | | 7% |  |
| Other | | | 7% | | | 9% | | | 6% | | 6% | | 5% | | 7% | | 5% | | 7% |  |
| Education | | |  | | |  | | |  | |  | |  | |  | |  | |  |  |
| <High school | | | 4% | | | 4% | | | 18% | | 22% | | 11% | | 22% | | 23% | | 10% |  |
| High school | | | 10% | | | 18% | | | 16% | | 28% | | 12% | | 22% | | 19% | | 26% |  |
| Some college | | | 28% | | | 25% | | | 26% | | 29% | | 21% | | 22% | | 33% | | 34% |  |
| College grad + | | | 58% | | | 54% | | | 40% | | 21% | | 56% | | 34% | | 25% | | 30% |  |
| **2018** | | | **Berkeley** | | | | | | **Oakland** | | | | **San Francisco** | | | | **Richmond** | | |  |
|  | | | City | | | Study | | | City | | Study | | City | | Study | | City | | Study |  |
| Race/ethnicity | | |  | | |  | | |  | |  | |  | |  | |  | |  |  |
| Black | | | 7% | | | 24% | | | 22% | | 39% | | 5% | | 21% | | 16% | | 35% |  |
| Hispanic/Latinx | | | 12% | | | 14% | | | 26% | | 47% | | 15% | | 46% | | 40% | | 34% |  |
| White | | | 52% | | | 44% | | | 30% | | 5% | | 40% | | 18% | | 19% | | 19% |  |
| Asian | | | 22% | | | 10% | | | 16% | | 3% | | 34% | | 9% | | 20% | | 6% |  |
| Other | | | 8% | | | 8% | | | 6% | | 6% | | 6% | | 7% | | 5% | | 6% |  |
| Education | | |  | | |  | | |  | |  | |  | |  | |  | |  |  |
| <High school | | | 4% | | | 4% | | | 17% | | 31% | | 10% | | 23% | | 18% | | 12% |  |
| High school | | | 11% | | | 17% | | | 16% | | 29% | | 13% | | 22% | | 22% | | 25% |  |
| Some college | | | 27% | | | 23% | | | 25% | | 23% | | 19% | | 19% | | 34% | | 30% |  |
| College grad + | | | 58% | | | 56% | | | 42% | | 16% | | 58% | | 35% | | 26% | | 33% |  |
| **2019** | | **Berkeley** | | | | | | **Oakland** | | | | | | **San Francisco** | | | | **Richmond** | | |
|  | | City | | | Study | | | City | | | | Study | | City | | Study | | City | | Study |
| Race/ethnicity | |  | | |  | | |  | | | |  | |  | |  | |  | |  |
| Black | | 7% | | | 22% | | | 22% | | | | 41% | | 5% | | 26% | | 18% | | 42% |
| Hispanic/Latinx | | 12% | | | 14% | | | 26% | | | | 40% | | 15% | | 37% | | 48% | | 29% |
| White | | 52% | | | 41% | | | 30% | | | | 7% | | 40% | | 23% | | 16% | | 15% |
| Asian | | 22% | | | 14% | | | 16% | | | | 3% | | 35% | | 8% | | 13% | | 8% |
| Other | | 7% | | | 9% | | | 6% | | | | 9% | | 5% | | 6% | | 6% | | 6% |
| Education | |  | | |  | | |  | | | |  | |  | |  | |  | |  |
| <High school | | 3% | | | 3% | | | 16% | | | | 22% | | 11% | | 14% | | 22% | | 9% |
| High school | | 11% | | | 17% | | | 16% | | | | 31% | | 13% | | 25% | | 23% | | 34% |
| Some college | | 26% | | | 23% | | | 24% | | | | 26% | | 19% | | 24% | | 27% | | 30% |
| College grad + | | 59% | | | 57% | | | 43% | | | | 21% | | 57% | | 37% | | 28% | | 27% |
| **2021** | | | **Berkeley** | | | | | | **Oakland** | | | | **San Francisco** | | | | **Richmond** | | | |
|  | | | City | | | Study | | | City | | Study | | City | | Study | | City | | Study | |
| Race/ethnicity | | |  | | |  | | |  | |  | |  | |  | |  | |  | |
| Black | | | 7% | | | 14% | | | 21% | | 45% | | 5% | | 35% | | 19% | | 36% | |
| Hispanic/Latinx | | | 12% | | | 20% | | | 26% | | 31% | | 16% | | 28% | | 40% | | 28% | |
| White | | | 52% | | | 41% | | | 30% | | 9% | | 37% | | 17% | | 21% | | 18% | |
| Asian | | | 22% | | | 13% | | | 16% | | 5% | | 35% | | 10% | | 13% | | 8% | |
| Other | | | 8% | | | 11% | | | 7% | | 10% | | 7% | | 9% | | 7% | | 10% | |
| Education | | |  | | |  | | |  | |  | |  | |  | |  | |  | |
| <High school | | | 3% | | | 2% | | | 15% | | 16% | | 10% | | 9% | | 17% | | 9% | |
| High school | | | 12% | | | 13% | | | 17% | | 31% | | 13% | | 30% | | 22% | | 28% | |
| Some college | | | 25% | | | 16% | | | 23% | | 29% | | 18% | | 27% | | 27% | | 24% | |
| College grad + | | | 59% | | | 69% | | | 46% | | 24% | | 59% | | 34% | | 34% | | 39% | |

City estimates are pulled from the United States Census Bureau’s American Community Survey 1-year estimates.

Supplemental Table 2. Sensitivity analysis for adjusted change in perceived consumption of soda, sports drink, and fruit drink consumption (times per day), with Berkeley as a comparison city

| **City** | **Time period** | **Adjusted change in perceived consumption,^a^**  **times per day (95% CI)** | | |  |
| --- | --- | --- | --- | --- | --- |
|  |  | **Soda** | **Sports drinks** | **Fruit drinks** | |
| Oakland^b^ | Tax implementation | -0.06 (-0.31, 0.20) | -0.36 (-0.61, -0.11)* | -0.16 (-0.41, 0.09) | |
| San Francisco^c^ | Ballot passing | 0.24 (-0.06, 0.53) | 0.19 (-0.09, 0.47) | 0.26 (0.01, 0.52)* | |
|  | Tax implementation | 0.12 (-0.13, 0.37) | 0.09 (-0.14, 0.33) | 0.09 (-0.12, 0.30) | |

^a^ Models adjust for gender, age, race/ethnicity, education, and SSB consumption, with a random intercept for neighborhood. Estimates reflect a comparison of differences in perceived consumption (how often respondents think others consume said beverages, times/day).

^b^ Oakland’s 2017-2021 post-tax implementation estimates are relative to 2016.

^c^ San Francisco’s 2017 post-ballot measure/pre-tax implementation estimates are relative to 2016, and the 2018-2021 estimates are the additional effect following the implementation of the tax.

* Results are significant; a 95% confidence interval does not include the null.

Supplemental Table 3. Sensitivity analysis for adjusted change in attitudes about the healthfulness of soda, sports drinks, and fruit drinks, with Berkeley as a comparison city

| **City** | **Time period** | **Adjusted change in attitudes about healthfulness of SSBs^a^**  **(95% CI)** | | |
| --- | --- | --- | --- | --- |
|  |  | **Soda** | **Sports drinks** | **Fruit drinks** |
| Oakland^b^ | Tax implementation | -0.16 (-0.36, 0.03) | -0.23 (-0.47, 0.01) | -0.15 (-0.40, 0.10) |
| San Francisco^c^ | Ballot passing | -0.10 (-0.33, 0.14) | -0.21 (-0.52, 0.10) | -0.15 (-0.47, 0.16) |
|  | Tax implementation | -0.19 (-0.38, 0.00) | -0.34 (-0.60, -0.08)* | -0.41 (-0.66, -0.15)* |

^a^ Models adjust for gender, age, race/ethnicity, education, and SSB consumption, with a random intercept for neighborhood. Estimates reflect a comparison of differences in attitudes about the healthfulness of soda, sports drinks, and fruit drinks, on a scale from 1 (extremely unhealthy) to 7 (extremely healthy).

^b^ Oakland’s 2017-2021 post-tax implementation estimates are relative to 2016.

^c^ San Francisco’s 2017 post-ballot measure/pre-tax implementation estimates are relative to 2016, and the 2018-2021 estimates are the additional effect following the implementation of the tax.

* Results are significant; a 95% confidence interval does not include the null.

Supplemental Figure 1. Adjusted marginal social norms (perceived consumption) of SSBs (soda, sports drinks, and fruit drinks) by city and year (times/day)


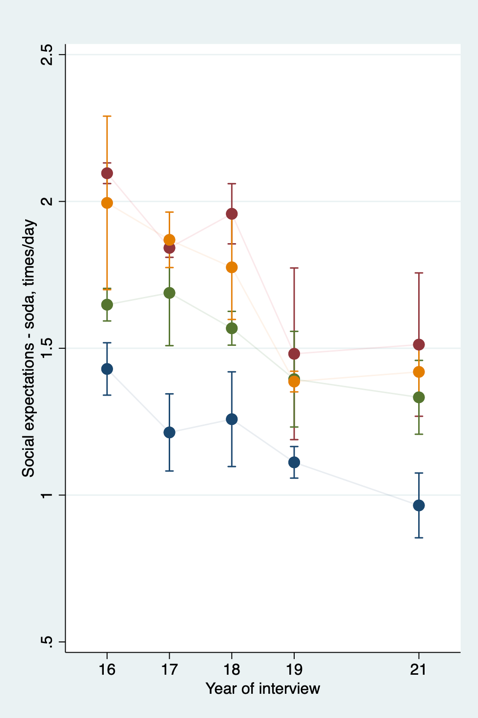

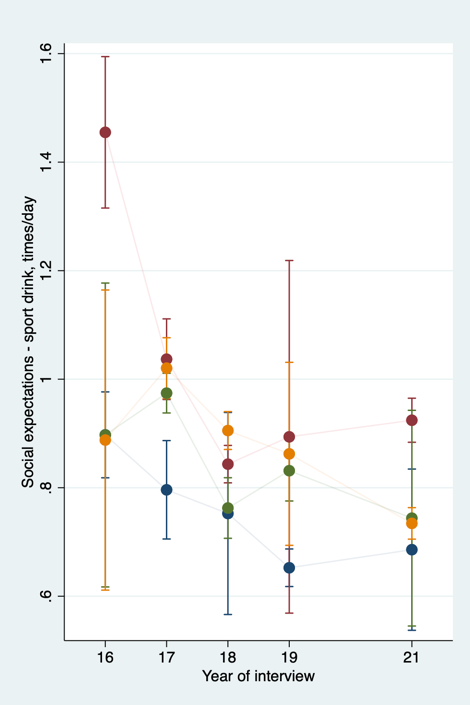

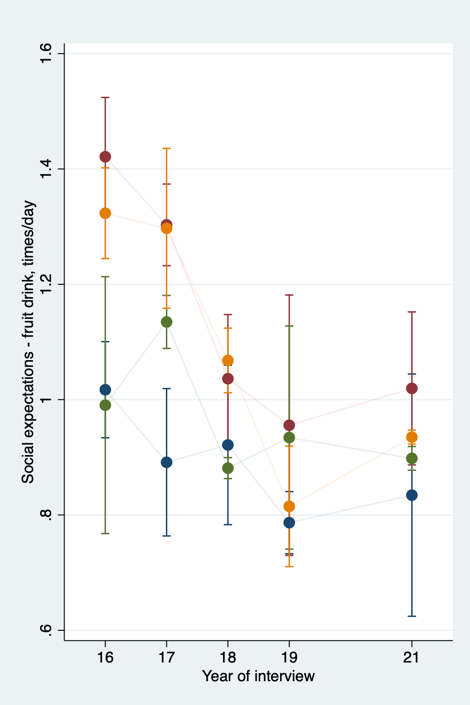


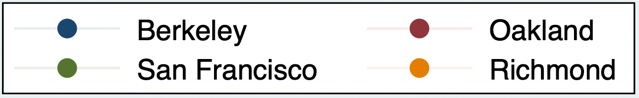


Models adjust for gender, age, race/ethnicity, education, and SSB consumption, with a random intercept for neighborhood and interaction between city and year as exposure. Estimates reflect adjusted marginal perceived consumption (how often respondents think others consume said beverages, times/day). Interaction between year and city is significant (p<0.001).

Supplemental Figure 2. Adjusted marginal attitudes about healthfulness of soda, sports drinks, and fruit drinks, by city and year


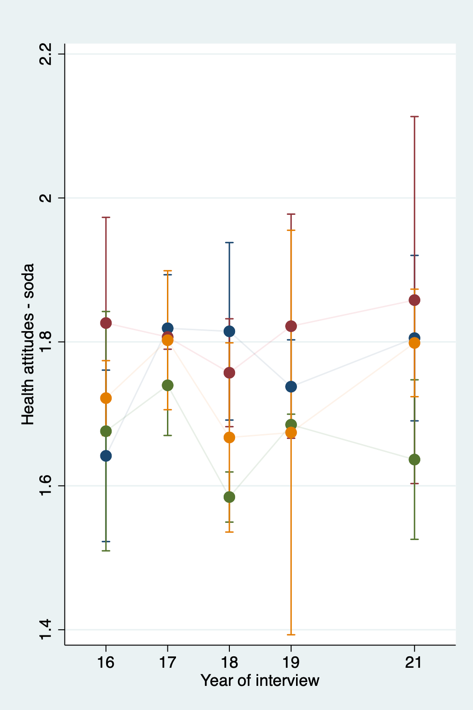

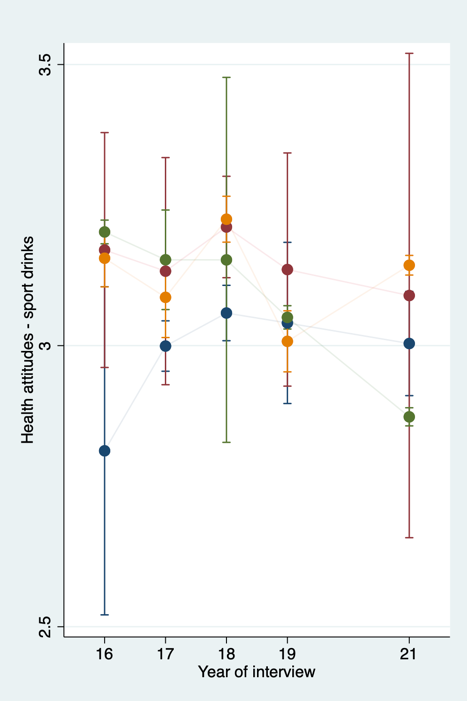

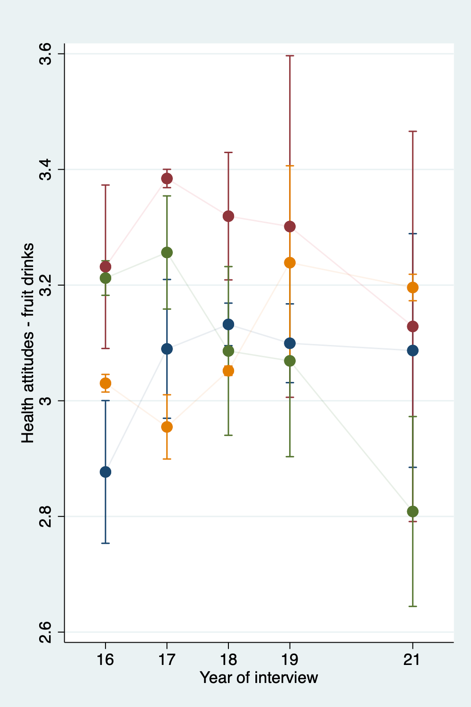


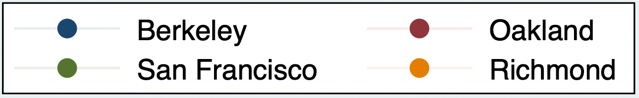


Models adjust for gender, age, race/ethnicity, education, and SSB consumption, with a random intercept for neighborhood and interaction between city and year as exposure. Estimates reflect adjusted mean attitudes about healthfulness of SSBs. Interaction between year and city is significant (p<0.001). Estimates are on a scale from 1 (extremely unhealthy) to 7 (extremely healthy).
